# Sexually transmitted infections related care-seeking behavior and associated factors among reproductive age women in Ethiopia: further analysis of the 2016 demographic and health survey

**DOI:** 10.1186/s12905-020-01145-9

**Published:** 2020-12-14

**Authors:** Simegnew Handebo

**Affiliations:** Department of Health Education and Behavioral Sciences, Institute of Public Health, College of Medicine and Health Sciences, University of Gondar, P.O.Box- 196, Gondar, Ethiopia

**Keywords:** Sexually transmitted infections, Care-seeking behavior, Women, Ethiopia

## Abstract

**Background:**

Sexually transmitted infections (STIs) are the most common communicable diseases that affect the health and life of people. Even though there is little information on the prevalence of STIs in Ethiopia, the problem is likely similar to other developing countries. Therefore, the objective of this study was to measure STIs related care-seeking behavior and associated factors among reproductive-age women in Ethiopia.

**Methods:**

The study was based on the data from the 2016 Ethiopian Demographic and Health Survey (EDHS). Information on STIs related care-seeking was extracted from the individual women dataset. A total of 474 (weighted) reproductive age women (15–49 years) who reported STIs or STI symptoms were included in the study. Bivariate and multivariable logistic regression models were fitted to assess factors associated with STIs related care-seeking behavior. The adjusted odds ratio (AOR) with the corresponding 95% confidence intervals (CI) was used to show the strength of associations between the outcome and independent variables. Variables with a *p* value of less than 0.05 were considered statistically significant.

**Results:**

The prevalence of STIs related care-seeking behavior among women was 33.3% (95% CI 29.2–37.3%). STIs related care-seeking behavior was significantly associated with higher women educational status (AOR = 0.16, 95% CI 0.03, 0.87), having a husband working an unskilled job (AOR = 6.99, 95% CI 1.34, 36.48), women who did not know their husband’s job (AOR = 12.79, 95% CI 2.24, 73.11), having an educated husband (AOR = 5.66, 95% CI 1.36, 23.51), being currently pregnant (AOR = 4.87, 95% CI 1.93, 12.28), being in the richer (AOR = 4.68, 95% CI 1.52, 14.39) and richest (AOR = 6.89, 95% CI 1.90, 24.81) wealth index.

**Conclusion:**

STIs related care-seeking behavior was significantly low among Ethiopian women. Surprisingly, STIs related care-seeking behavior was lower among an educated woman. In contrast, having an educated husband, women who didn’t know their husband’s job, a husband working an unskilled job, being pregnant, and high wealth status were positively associated with STIs related care-seeking behavior.

## Background

Sexually transmitted diseases are various clinical syndromes and infections caused by pathogens that will be acquired and transmitted through sexual contact [1]. STIs are the major public health problem worldwide which affects the quality of life of the people and causes serious morbidity and mortality. Chlamydia and gonorrhea cause serious complications like pelvic inflammatory disease, ectopic pregnancy, infertility, chronic pelvic pain and arthritis [2]. Besides syphilis causes neurological, cardiovascular and dermatological disease in adults, while stillbirth, neonatal death, premature delivery or severe disability in infants [2–5]. Each year it is estimated that 200,000 fetal and neonatal deaths occur due to syphilis in the pregnancy and over 280,000 cervical cancer deaths happen due to human papillomavirus (HPV) [6]. Untreated STIs facilitate the transmission and acquisition of human immunodeficiency virus (HIV) [7]. Globally, these infections constitute huge health and economic burden, particularly in developing countries where they account for 17% of economic losses due to ill-health [3, 5, 8, 9].

STIs prevention and control have widespread benefits and contribute to the achievement of Sustainable Development Goals related to ending under five children deaths, combating communicable diseases, and providing sexual and reproductive health care [6]. In 2016, World Health Organization (WHO) released Global Health Sector Strategy on STIs with the goal of ending STIs epidemics in the period of 2016–2021 [10]. Each strategy defines the global targets depending on strong disease surveillance systems for monitoring the progress. This WHO Global Health Sector Strategy on STI includes the subsequent targets to be achieved by 2030: a 90% reduction of syphilis incidence, a 90% reduction in gonorrhea incidence, and 50 or fewer cases of congenital syphilis per 100,000 live births in 80% of countries [11].

The burden of STIs on the health care system and expenditure is large. Even excluding HIV, STIs are consistently amongst the most common conditions leading to health care visits. In developing countries, STIs are the leading causes of disability-adjusted life years lost for reproductive age women, subsequent to maternal causes and HIV. In all nations, particularly in developing countries, STIs result in substantial productivity losses on individuals and communities [12]. It disproportionately affects the health and social well-being of women by producing a significant impact on their reproductive potential [13, 14].

In Ethiopia, many factors hinder women from obtaining medical advice or treatment when they are sick. About eight in ten women (78%) live in rural areas. More than 2 in 3 women (70%) report having at least one barrier in accessing health care. Lack of money (55%), distance from a health facility (50%), not wanting to go alone (42%), and getting permission to go for treatment (32%) are some of the main barriers in accessing health care services. In the country, 9.5% and 40.3% of young age women (15–24) start sexual intercourse before age of 15 and 18 years old, respectively [15]. A significant proportion of the people, particularly youths has been identified as at risk of STIs [16, 17]. A high prevalence of STIs symptoms is observed among individuals who seek health care from health institutions [14]. On the other hand, most people with STIs symptoms did not seek care from any source [18].

Prompt identification and treatment is a cornerstone of STIs prevention and control; reduces the prevalence and breaks the chain of transmission [12]. In many low and middle-income countries, health services did not fully address the sexual and reproductive health (SRH) needs [19, 20]. Understanding STIs related care-seeking behavior of women inform policies and strategies aimed at improving the accessibility and acceptability of STIs care services. Most people with STIs do not seek treatment at public health facilities as they have minor or no symptoms while others take self-prescribed drugs. Women with self-reported symptoms of sexual morbidity do not seek treatment due to taboos and inhibitions regarding sexual and reproductive health [21]. Therefore, the objective of this study was to measure STIs related care-seeking behavior and associated factors among reproductive-age women in Ethiopia.

## Methods

### Study area

The study was conducted in Ethiopia, which is the second most populous nation in Africa after Nigeria, and the fastest growing economy in the region. The total population of the country was projected to be 109 million in 2018 [22]. Ethiopia is a land of enormous diversity with more than 80 languages. The major health problems of the country remain largely preventable communicable diseases and nutritional disorders. The country is administratively subdivided into nine regional states and two city administrations.

### Data source and study subjects

This study used data from the 2016 EDHS which is publicly available from the Measure DHS website (http://www.measuredhs.com). The 2016 EDHS is the fourth and the most recent population-based study conducted across the country (in nine regions [Tigray, Afar, Amhara, Oromia, Somali, Benishangul-Gumuz, Southern Nations, Nationalities and Peoples’ Region [SNNPR], Gambella and Harari] and two cities [Addis Ababa and Dire Dawa]). It used a two-stage cluster sampling design with rural–urban and regions as strata. In the first stage, enumeration areas (EAs) were selected using probability proportional to the size of EAs. In the second stage, a random sample of 18,008 households and 16,583 reproductive women age 15–49 were selected. A total of 15,683 women aged 15–49 years were interviewed, yielding a response rate of 95%. The study used the dataset from the individual women in this analysis. The study was conducted from January 18, 2016, to June 27, 2016. Furthermore, selections of households, validation procedures, and data quality assurance are available in detail elsewhere [15].

### Sample size

Of the total 15,683 women included in the 2016 EDHS, 474 (weighted) who reported STIs or symptoms of STIs in the 12 months preceding the survey were analyzed.

### Outcome variable

In the 2016 EDHS women respondents whoever had sex were asked whether they had STIs or symptoms of STIs (a bad-smelling, abnormal discharge from the vagina or a genital sore or ulcer) in the 12 months before the survey. “Yes” was selected for respondents who had STIs or STI symptoms and “no” otherwise. A further question on having sought treatment or advice was posed to the respondents who reported STIs or STIs symptoms. “Yes” was selected for respondents who sought treatment and/or advice and “no” otherwise. The study adopted the response to this question to assess STIs related care-seeking behavior and associated factors among reproductive-age women in Ethiopia. The description and measurement of the outcome variables used in the study are presented in Table 1.

**Table 1:** Description and measurements of STIs related care seeking behavior

Variable	Description	Measurements
*Outcome variable*		
Had STI infection	Have you had STI or symptoms of an STI (a bad-smelling, abnormal discharge from the vagina or a genital sore or ulcer) in the 12 months before the survey?	“Yes” = respondents who had STIs or STI symptoms and “no” otherwise
STIs related care-seeking behavior (outcome)	If you had the infection, did you seek any kind of advice or treatment?	“Yes” = for respondents who sought treatment or advice and “no” otherwise

### Explanatory variable

Based on literature [18, 20–23] age, marital status (never married, married, formerly married), place of residence (urban and rural), educational status (no education, primary, secondary, higher), religion (Orthodox, Muslim, Protestant, others), occupational status (not working, professional, agricultural, unskilled, others), husband occupation (not working, professional, agricultural, unskilled, others), husband educational status (no education, primary, secondary, higher and don’t know), administrative region (Tigray, Affar, Amhara, Oromia, Benishangul-gumuz, Southern Nations, Nationalities, and People’s Region (SNNPR), Gambela, Harari, Somali, Dire Dawa, Addis Ababa), wealth index (Poorer, Poorest, Middle, Richer, and Richest), media exposure (reading newspaper, listening to the radio, and watching television), currently working, pregnancy status, ever heard about STI, and ever tested for HIV were the selected independent variables.

### Statistical analysis

The data were analyzed using STATA version 12 (Stata Corporation, College Station, TX, USA). A sampling weight was applied in all analyses to adjust for the non-proportional allocation of the sample to different regions, urban and rural areas, and for the possible differences in the response rates. Hence, the actual representativeness of the survey results both at the national and regional levels was ensured. Summary statistics were conducted to describe the characteristics of the study participants. Bivariate and multivariable logistic regression analyses were conducted to identify factors associated with STIs related care-seeking behavior. All variables with a *p* value of ≤ 0.2 in the bi-variable logistic regression analysis were entered into the multivariable analysis. Adjusted odds ratio (AOR) with the corresponding 95% CI was used to show the strength of associations between the outcome and the independent variables. Variables with a *p* value of less than 0.05 were considered as statistically significant.

## Results

### Sociodemographic characteristics of participants

A total of 474 (weighted) reproductive age women (15–49 years) who had STIs or STIs symptoms were included in the study. There were no missing values in the data. The mean age of the women was 31.5 (SD ± 0.4) years. The majority of 384 (73.4%) the respondents were rural residents and 387 (81.8%) were married. Nearly half of women (49.6%) had no education and about 50.9% were Orthodox Christian followers. More than one-third 163 (34.5%) were from the Oromia region and followed by 147 (31.0%) Amhara region. Regarding the wealth index, 157 (33.1%) of women were in the richest wealth quintile (Table 2).

**Table 2:** Socio-demographic characteristics of reproductive age women reported STIs or STIs symptoms, EDHS 2016

Characteristics	Percentage of care-seeking behavior	Weighted frequency (%)
*Age of women (years)*		
15–19	30.3	21 (4.4)
20–24	26.1	76 (16.0)
25–29	46.1	105 (22.2)
30–34	35.9	74 (15.7)
35–39	23.9	86 (18.2)
40–44	31.5	81 (17.1)
45–49	33.9	31(6.5)
*Residence*		
Urban	53.2	126 (26.5)
Rural	26.1	384 (73.4)
*Marital status*		
Never married	47.4	26 (5.4)
Married	30.0	387 (81.8)
Formerly married	48.5	61 (12.8)
*Administrative region*		
Tigray	34.1	39 (8.2)
Afar	17.0	3 (0.6)
Amhara	30.6	147 (31.0)
Oromia	29.7	163(34.5)
Somali	48.8	17 (3.6)
Benishangul Gumuz	46.2	2 (0.4)
SNNPR	33.9	73 (15.5)
Gambela	36.4	1.4 (0.3)
Harari	19.8	0.54(0.1)
Addis Ababa	60.0	25(5.3)
Dire dawa	34.1	2.5(0.5)
*Educational status of the women*		
No education	24.0	235 (49.6)
Primary	39.2	150 (31.7)
Secondary	55.2	35 (7.4)
Higher	42.7	54 (11.3)
*Religion*		
Orthodox	34.0	241 (50.9)
Muslim	33.3	154.6 (32.6)
Protestant	33.6	66.6 (14.1)
Other	17.4	11.5 (2.43)
*Women occupation*		
Not working	25.6	190 (40.1)
Professional	48.1	146 (30.8)
Agriculture	26.5	126 (26.6)
Unskilled	50.4	6 (1.3)
Don't know	41.4	6 (1.3)
*Husband Occupation*		
Not working	12.0	45.7 (11.8)
Professional	54.4	102.3 (26.4)
Agriculture	17.5	211.3 (54.5)
Unskilled	43.9	11.1 (2.9)
Don't know	76.5	17 (4.4)
*Husband education*		
No education	22.6	169 (43.7)
Primary	21.8	132 (34.2)
Secondary	47.5	37 (9.5)
Higher	61.3	43 (11.2)
Don’t know	89.6	5 (1.4)
*Wealth index*		
Poorest	13.5	77.8 (16.4)
Poorer	21.6	83 (17.5)
Middle	20.5	73.5(15.5)
Richer	40.4	83 (17.5)
Richest	51.5	157 (33.1)

### Reproductive health and communication characteristic

Among the study participant, 434 (91.6%) ever heard about STIs. The majorities of women 259 (54.6%) were ever tested for HIV. More than two-third 330 (69.7%) of the women were sexually active in the last 4 weeks preceding the interview. The majority of participants 393 (82.9%) have a single sexual partner (Table 3). With regard to communication, 19.9%, 16.5%, and 5.9% of the participant listened to the radio, watched television, and read newspapers at least once a week, respectively.

**Table 3:** Reproductive health characteristics of reproductive age women reported STIs or STIs symptoms, EDHS 2016

Characteristics	Percentage of care-seeking behavior	Weighted frequency (%)
*Ever heard about STIs*		
No	7.8	40 (8.4)
Yes	35.6	434 (91.6)
*Ever heard about AIDS*		
No	7.4	42 (8.9)
Yes	35.8	432 (91.1)
*Ever been tested for HIV*		
No	20.3	215 (45.4)
Yes	44.1	259 (54.6)
*Had a pre-marital HIV testing*		
No	23.8	285 (73.5)
Yes	47.0	103 (26.5)
*Recent sexual activity*		
Active in last 4 weeks	30.0	330 (69.7)
Not active in last 4 weeks	40.8	144 (30.3)
*Condom used during last sex*		
No	31.1	394 (99.1)
Yes	40.1	4 (0.9)
*Number of sexual partners in the last 12 months*		
0	44.4	76 (16.0)
1	30.7	393 (82.9)
2	61.2	5 (1.0)
Don’t know	0	0.15(0.03)

### Prevalence of care-seeking behavior for STI

The prevalence of STIs related care-seeking behavior among women was 33.3% (95% CI 29.2–37.3%) in the past 12 months. This prevalence was 46.1% among the age group 25–29 years and 47% among women who had premarital HIV tests in the past 12 months. Care-seeking behavior varied across the regions in the country with the highest prevalence (60.0%) in Addis Ababa and the lowest in the Afar region (17.0%) in the past 12 months.

### Factors associated with care-seeking behavior for STI

In the multivariable logistic regression women education, husband’s educational status, husband’s occupation, wealth index, and pregnancy status were significantly associated (*p* < 0.05) with STIs related care-seeking behavior. The odds of STIs related care-seeking behavior of the women with a higher education decreased by 84% (AOR = 0.16, 95% CI 0.03, 0.87) compared to those with no education. The odds of STIs related care-seeking behavior of the women whose husbands were working an unskilled job (AOR = 6.99, 95% CI 1.34, 36.48) and who did not know their husband’s job (AOR = 12.79, 95% CI 2.24, 73.11) were higher than those who had an unemployed husband. The odds of STIs related care-seeking behavior of women who had an educated husband was 5.66 (AOR = 5.66, 95% CI 1.36, 23.51) higher than those whose husbands had no education. For the women in the richer and richest wealth status, the odds of STs related care-seeking behavior were 4.68 (AOR = 4.68, 95% CI 1.52, 14.39) and 6.89 (AOR = 6.89, 95% CI 1.90, 24.81) times higher compared to those in the poorest wealth quartile. The odds of STIs related care-seeking behavior of women who were currently pregnant was 4.87 (AOR = 4.87, 95% CI 1.93, 12.28) higher than non-pregnant women (Table 4).

**Table 4:** Factors associated with STIs related care-seeking behavior for among reproductive age women in Ethiopia, EDHS 2016

Explanatory variable	Care-seeking behavior for STIs	
	Crude OR (95% CI)	Adjusted OR (95% CI)
*Residence*		
Urban	1	1
Rural	0.31 (0.20, 0.47)	1.18 (0.36, 3.84)
*Educational status*		
No education	1	1
Primary	2.05 (1.32, 3.20)	1.08 (0.54, 2.18)
Secondary	3.90 (1.88, 8.08)	1.19 (0.34, 4.16)
Higher	2.36 (1.27, 4.39)	0.16 (0.03, 0.87)*
*Women currently working*		
Yes	1	1
No	1.48 (1.00, 2.18)	0.79 (0.42, 1.47)
*Husband occupation*		
Not working	1	1
Professional	8.71 (3.30, 23.02)	3.93 (1.27, 12.18)
Agriculture	1.55 (0.59, 4.04)	1.00 (0.33, 3.00)
Unskilled	5.72 (1.30, 25.16)	6.99 (1.34, 36.48)*
Don’t know	23.73 (5.67, 99.38)	12.79 (2.24,73.11)**
*Husband education*		
No education	1	1
Primary	0.95 (0.55, 1.65)	0.79 (0.38, 1.63)
Secondary	3.09 (1.47, 6.49)	0.92 (0.31, 2.73)
Higher	5.41 (2.67, 10.99)	5.66 (1.36, 23.51)*
Don’t know	29.39 (1.75, 493.43)	18.37 (0.68, 498.00)
*Wealth index*		
Poorest	1	1
Poorer	1.76 (0.76, 4.05)	2.97 (0.95, 9.29)
Middle	1.65 (0.70, 3.91)	2.90 (0.95, 8.88)
Richer	4.34 (1.98, 9.50)	4.68 (1.52, 14.39)**
Richest	6.78 (3.30, 13.94)	6.89 (1.90, 24.81)**
*Frequency of watching television*		
Not at all	1	1
Less than once a week	1.76 (1.03, 3.01)	1.90 (0.85, 4.28)
At least once a week	3.57 (2.14, 5.95)	1.51 (0.48, 4.74)
*Frequency of reading newspaper*		
Not at all	1	1
Less than once a week	2.80 (1.54, 5.09)	0.62 (0.22, 1.77)
At least once a week	1.98 (0.91, 4.30)	0.62 (0.15, 2.64)
*Ever heard about STIs*		
No	1	1
Yes	6.58 (2.03, 21.34)	11.98 (1.25, 115.13)
*Ever been tested for HIV*		
No	1	1
Yes	3.10 (2.05, 4.69)	1.15 (0.62, 2.14)
*Currently pregnant*		
Yes	1	1
No	1.77(0.89, 3.49)	4.87 (1.93, 12.28)**

^*^Significant at *p* < to 0.05; **significant at *p* < to 0.01

## Discussion

The study revealed that the STIs related care-seeking behavior of the women reported STIs was relatively low. In this study among women who reported STIs, only 33.3% of them sought treatment or advice in the past 12 months. This finding is comparable with the EDHS 2011 finding and study done among Ghanaian Women in Accra city [23, 24]. This might be due to many women who experience STIs symptoms do not regard them as serious and do not believe that they need to be treated [25]. However, it was much lower than studies conducted in Kenya, Malawi, Nigeria, and Tamil Nadu district in India [21, 25–27]. The difference might be due to the differences in knowledge, access to health facilities, and socio-economic factors.

STIs related care-seeking behavior is influenced by a different group of factors [21]. In this study, women education, husband’s educational status, husband’s occupation, wealth index, and pregnancy status were factors associated with STIs related care-seeking behavior. Despite the fact that education has a valuable input in enhancing female’s confidence and capability to make decisions about their own health, surprisingly in this study higher women’s education was associated with lower care-seeking behavior compared to those with no education. This may perhaps be due to greater confidence in the ability to manage the illnesses at the home level or due to other confounding factors in those with further education. On the other hand, it may be due to literacy acquired outside of formal education among illiterate women that may lead them to had good care-seeking behavior. Other studies found that higher women education positively influences care-seeking behavior [21, 28].

Husband’s education also shows a significant association with STI related care-seeking behavior among women in Ethiopia. The women who had more highly educated husbands have higher odds of seeking STI related healthcare than women who have husbands with no education. This finding is consistent with the study conducted in India, in which women of higher educated husbands have higher care-seeking behavior than women of illiterate husbands [28]. This might be due to husbands with a higher level of education allow their wives to be autonomous and support them in seeking health care services. Similarly, STIs related care-seeking behavior of women with a husband working an unskilled job and who did not know their husband’s job was higher compared to women who have an unemployed husband.

Economic status was also the other significant factor associated with women’s care-seeking behavior. Women in the richer and richest wealth quintile level were more likely to seek treatment than those women in the poorest wealth quintile. This finding highlights the complex relationship between economic status and health care service utilization. This means the lower wealth quintile is associated with reduced chances that the women would seek care. This finding was supported by that of studies conducted in Ghana, Nigeria, India, and Tamilnadu state in India [21, 23, 27, 28]. This might be due to women who have a good economic status able to overcome financial barriers to access health care services. On the other hand, pregnant women were more likely to seek treatment than non-pregnant women. The possible explanation is that pregnant women get counseling and awareness of STIs and early treatment during antenatal care visits.

The main strength of this study was it used nationally representative community-based data. But this study also has a few limitations that should be mentioned. Firstly, care-seeking data is based on the women’s report which can have potential recall bias or information error. Secondly, we didn’t include information about partner STI care-seeking. The other was a lot of the factors associated with care-seeking presented in the results have very wide CIs due to the relatively small number of women who reported STIs.

## Conclusion

This study showed that the STIs related care-seeking behavior still remains poor among women in Ethiopia. Surprisingly, STIs related care-seeking behavior was lower among an educated woman. In contrast, having an educated husband, not knowing a husband’s job, having a husband working an unskilled job, being pregnant, and high wealth status were positively associated with STIs related care-seeking behavior. Therefore, targeted interventions need to be developed to increase prompt care-seeking behavior among women in Ethiopia. Further researches are suggested on the STIs related care-seeking behavior with a large sample size and incorporating factors like the accessibility of health facilities, women empowerment, and qualitative approach.

## Data Availability

The raw data used in this study can be accessed from the DHS website: http://www.dhsmeasures.

## Abbreviations

AOR: Adjusted odds ratio
CI: Confidence interval
CSA: Central Statistical Agency
EAs: Enumeration areas
EDHS: Ethiopia Demographic and Heath Surveys
HIV: Human immunodeficiency virus
HPV: Human papilloma virus
SD: Standard deviation
SNNPR: Southern Nations, Nationalities, and People’s Region
STIs: Sexually transmitted infections
WHO: World Health Organization
